# Suicide in Older Adult Men Is Not Related to a Personal History of Participation in Football

**DOI:** 10.3389/fneur.2021.745824

**Published:** 2021-11-26

**Authors:** Grant L. Iverson, Amy Deep-Soboslay, Thomas M. Hyde, Joel E. Kleinman, Brittany Erskine, Amanda Fisher-Hubbard, Joyce L. deJong, Rudolph J. Castellani

**Affiliations:** ^1^Department of Physical Medicine and Rehabilitation, Harvard Medical School, Boston, MA, United States; ^2^Spaulding Rehabilitation Hospital, Charlestown, MA, United States; ^3^Spaulding Research Institute, Charlestown, MA, United States; ^4^MassGeneral Hospital for Children Sports Concussion Program, Boston, MA, United States; ^5^Home Base, A Red Sox Foundation and Massachusetts General Hospital Program, Charlestown, MA, United States; ^6^Lieber Institute for Brain Development, Johns Hopkins Medical Campus, Baltimore, MD, United States; ^7^Department of Psychiatry & Behavioral Sciences, Johns Hopkins School of Medicine, Baltimore, MD, United States; ^8^Department of Neurology, Johns Hopkins School of Medicine, Baltimore, MD, United States; ^9^Department of Pathology, Western Michigan University Homer Stryker M.D. School of Medicine, Kalamazoo, MI, United States; ^10^Department of Pathology, Northwestern University Feinberg School of Medicine, Chicago, IL, United States

**Keywords:** suicide, concussion, autopsy, sports, depression

## Abstract

**Introduction:** It is reasonable to estimate that tens of millions of men in the United States played high school football. There is societal concern that participation in football confers risk for later-in-life mental health problems. The purpose of this study is to examine whether there is an association between a personal history of playing high school football and death by suicide.

**Methods:** The subjects were obtained from the Lieber Institute for Brain Development (LIBD) brain donation program in collaboration with the Office of the Medical Examiner at Western Michigan University Homer Stryker MD School of Medicine. Donor history was documented via medical records, mental health records, and telephone interviews with the next-of-kin.

**Results:** The sample included 198 men aged 50 or older (median = 65.0 years, interquartile range = 57–75). There were 34.8% who participated in contact sports during high school (including football), and 29.8% participated in high school football. Approximately one-third of the sample had suicide as their manner of death (34.8%). There was no statistically significant difference in the proportions of suicide as a manner of death among those men with a personal history of playing football compared to men who did not play football or who did not play sports (*p* = 0.070, Odds Ratio, OR = 0.537). Those who played football were significantly less likely to have a lifetime history of a suicide attempt (*p* = 0.012, OR = 0.352). Men with mood disorders (*p* < 0.001, OR = 10.712), substance use disorders (*p* < 0.020, OR = 2.075), and those with a history of suicide ideation (*p* < 0.001, OR = 8.038) or attempts (*p* < 0.001, OR = 40.634) were more likely to have suicide as a manner of death. Moreover, those men with a family history of suicide were more likely to have prior suicide attempts (*p* = 0.031, OR = 2.153) and to have completed suicide (*p* = 0.001, OR = 2.927).

**Discussion:** Suicide was related to well-established risk factors such as a personal history of a mood disorder, substance abuse disorder, prior suicide ideation, suicide attempts, and a family history of suicide attempts. This study adds to a steadily growing body of evidence suggesting that playing high school football is not associated with increased risk for suicidality or suicide during adulthood.

## Introduction

Researchers have reported that former amateur and professional football players are at risk for chronic traumatic encephalopathy (CTE) (1), and that 99% of former National Football League (NFL) players whose brains were donated for research had mild or greater CTE neuropathologic change (2). Some researchers have asserted that suicidality and suicide are clinical features of CTE (1, 3–8). The proposed association between suicide and CTE is controversial because (i) for 80 years, between 1929 and 2009, suicide was not reported to be a manner of death or a clinical feature of CTE in the published medical literature (9–11); (ii) epidemiological studies involving causes of death in former NFL players, published in 2012 (12) and 2016 (13), illustrated that their rate of suicide was approximately *half* the rate of men from the general population; and (iii) to our knowledge there are no published case-control, cross-sectional, cohort, longitudinal, or epidemiological studies that have shown such an association between CTE neuropathologic change and suicide. Victoroff reviewed the literature and proposed research criteria for traumatic encephalopathy syndrome, and he did not include suicidality in his criteria (11). Moreover, authors of five review papers, published between 2013 and 2020, concluded that the scientific evidence to support the theory that suicide is a clinical feature of CTE is lacking (14–18).

Given that football has been one of the most popular sports in the United States for generations, and more than 1 million youth participated in high school football each year between 2000 and 2019 (19), it is reasonable to estimate that tens of millions of men in the United States have played high school football. Therefore, it is important to understand whether former high school football players are at increased risk for suicidality and suicide. Three studies (20–22) have used data from the National Longitudinal Study of Adolescent to Adult Health to examine whether playing high school football is associated with suicidality and other mental health problems 5 and 15 years later, during their early and late 20s. Those researchers reported that playing high school football was not associated with lifetime rates of depression (20–22), current symptoms of depression [i.e., within the past seven days (21, 22)], lifetime rates of anxiety (21), suicidal ideation within the past year (20–22), or substance abuse (i.e., nicotine, cannabis, alcohol) (21). Similarly, in a study of older adults, using data from the Wisconsin Longitudinal Study, playing high school football was not associated with depression when these men were ~65 years old (23).

The purpose of this study is to examine whether there is an association between a personal history of playing football and death by suicide. For the present study, the sample was obtained from the brain donation program at the Lieber Institute for Brain Development, which works with medical examiner offices to procure brain tissue from decedents who had neuropsychiatric problems in order to elucidate the molecular mechanisms associated with these illnesses. Researchers from the institute conduct a telephone interview with the next-of-kin to collect detailed demographic and clinical histories. We hypothesized that there would be no association between participation in football or contact sports and suicide. For this study, we categorize men into groups based on whether they played football, other contact sports, or no contact sports. We hypothesized that factors known to be associated with suicide, such as a personal history of depression or bipolar disorder (24–26), suicidality (27), substance abuse (28–30), and a family history of suicidality and suicide (31–33) would be associated with suicide in the present sample.

## Materials and Methods

### Participants

The subjects were obtained from the Lieber Institute for Brain Development (LIBD) brain donation program. Postmortem brain samples were donated to the LIBD from the Office of the Medical Examiner of the Western Michigan University Homer Stryker M.D. School of Medicine, Department of Pathology (WIRB protocol #1126332), with the informed consent of legal next-of-kin at the time of autopsy. An important feature of this brain donation program is the inclusion of a substantial percentage of people who completed suicide. Potential donors are identified each morning by reviewing information received from death scene investigations in the Western Michigan catchment area (13 counties in western Michigan) that took place in the preceding 24 h. Decedents who require forensic autopsy, as determined by a board-certified forensic pathologist, are screened for exclusionary criteria. The exclusionary criteria include: not-at-fault motor vehicle accidents, homicides, anything suspicious otherwise (e.g., elder abuse, child abuse, deaths in custody), specific infectious illnesses (hepatitis, human immunodeficiency virus, meningitis, sepsis, prion disease), structural brain damage (ischemic strokes, brain tumors, brain surgery history, cerebral palsy, or past severe traumatic brain injury), encephalopathy, seizure disorder without a psychiatric history, advanced life support for more than 24 h, last known to be alive more than 72 h prior, and evidence of decomposition.

Next-of-kin of decedents determined acceptable for referral are then contacted by LIBD to request consent for donation. One study documented that more than half (57.0%) of all African-American families and more than two-thirds (74.1%) of all Caucasian families who were contacted by the LIBD at the time of autopsy for consent to donate their deceased loved one's brain for neuropsychiatric research agreed to do so (34). Donor history is documented via medical records, mental health records, and telephone interviews with the next-of-kin (described below). Our subjects were all men aged 50 or greater because the purpose of the study was to examine the association between playing amateur football and death by suicide in older adult men.

### Manner of Death

All cases were reviewed in a multidisciplinary conference prior to autopsy and/or brain procurement. Manner of death was determined by a board-certified forensic pathologist in each case, according to guidelines from the National Association of Medical Examiners (35). The manner of death is an opinion based on an evaluation of all relevant information, including death scene investigation, information from witnesses, family members, and acquaintances, a review of relevant medical records, and a postmortem examination conducted by the forensic pathologist. Deaths are classified as suicide when an intentional self-inflicted injury or poisoning results in death. The manner of death and cause of death for the decedents is reported in Table 1.

**Table 1:** Characteristics of the sample.

Age	*M* = 66.41, SD = 11.31
Race (white); *n*, %	195, 98.5%
Education; *n*, %	
Fewer than 9 years	14, 7.1%
9–11 years	20, 10.1%
High school diploma or equivalent	75, 37.9%
Some college	39, 19.7%
Bachelor's degree	29, 14.6%
Graduate degree	14, 7.1%
Unknown or missing	7, 3.5%
Marital status; *n*, %	
Married	97, 49.0%
Divorced	49, 24.7%
Single	29, 14.6%
Separated	4, 2.0%
Widowed	18, 9.1%
Missing	1, 0.5%
Manner of death; *n*, %	
Accident; *n*, %	62, 31.3%
Natural causes; *n*, %	61, 30.8%
Suicide; *n*, %	69, 34.8%
Undetermined, *n*, %	6, 3.0%
Causes of death	
Cardiovascular disease, MI, CHF, cardiomyopathy	54, 27.3%
Alcohol, illicit drugs, or poly-drug related	22, 11.1%
Gunshot wound(s)	43, 21.7%
Hanging	11, 5.6%
Head/neck/spine injury	7, 3.5%
Blunt force injuries	7, 3.5%
Multiple injuries, MVA, chest injuries	15, 7.6%
Asphyxia	6, 3.0%
Drowning	11, 5.6%
Electrocution or thermal injuries	3, 1.5%
Pulmonary embolism, pneumonia, COPD, respiratory failure	9, 4.5%
Carbon monoxide poisoning	3, 1.5%
Other natural causes	7, 3.5%
Suicide ideation; *n*, %, missing (*n*)	64, 32.3%, 30
Suicide attempts; *n*, %, missing (*n*)	50, 25.3%, 21
Participation in contact sports; *n*, %, missing (*n*)	69, 34.8%, 0
Participation in football; *n*, %, missing (*n*)	59, 29.8%, 0
Traumatic brain injury; *n*, %, missing (*n*)	53, 26.8%, 10
Depression or bipolar disorder; *n*, %, missing (*n*)	100, 50.5%, 17
Substance use disorder; *n*, %, missing (*n*)	93, 47.0%, 15
Family psychiatric history; *n*, %, missing (*n*)	128, 64.6%, 23
Family history of suicide; *n*, %, missing (*n*)	53, 26.8%, 19

*M, mean; SD, Standard Deviation; n, sample size. For all subjects, it was known whether they were classified as having suicide as a manner of death (i.e., yes or no). For 6 people, the manner of death was not suicide, but the specific manner and cause(s) were not recorded in the database. Causes of death for those 6 were recorded as drowning for 2, hypothermia for 1, a MVA for 1, and drug intoxication for 2. MI, myocardial infarction; CHF, congestive heart failure; MVA, motor vehicle accident; COPD, chronic obstructive pulmonary disease*.

### Demographic and Clinical Information

At the time of donation, a 36-item next-of-kin informant telephone interview was conducted to obtain medical, social, demographic, family, and psychiatric history. During the phone interview, next-of-kin were asked whether the decedent had a history of head trauma. This was coded as yes or no and it was accepted as yes if there was a personal history of concussions in sports, head injury in daily life, or what clearly sounded like a severe traumatic brain injury. The family members described a broad range of mechanisms of injury (e.g., sports, car accidents, and falls from a height) and severity injury (e.g., concussion, skull fractures, subdural hematomas, and coma). It is assumed that all levels of brain injury severity, mild, moderate, and severe, are included in this binary classification, but for most cases there was insufficient information to classify brain injury severity. For most subjects, detailed notes on this variable were not available and it was coded as binary. Next-of-kin were also asked whether the decedent had a history of engagement in sports with a high risk of repetitive brain injury, including boxing, football, mixed martial arts, lacrosse, rugby, or auto racing, and this variable was coded as yes or no. We categorized men into groups based on whether they played football, other contact sports, or no contact sports.

A retrospective clinical diagnostic review was conducted on every brain donor, consisting of the telephone screening, autopsy, and forensic investigative data, forensic toxicology data, extensive psychiatric treatment, substance abuse treatment, and/or medical record reviews, and whenever possible, family informant interviews. All data were compiled into a comprehensive psychiatric narrative summary that was reviewed by two board-certified psychiatrists in order to arrive at lifetime DSM-5 psychiatric diagnoses and medical diagnoses. A subgroup of decedents were recruited who were free from psychiatric and substance use diagnoses, and their toxicological data was negative for drugs of abuse. Every brain donor had forensic toxicological analysis, which typically covered ethanol and volatiles, opiates, cocaine/metabolites, amphetamines, and benzodiazepines. Some donors also received supplemental directed toxicological analysis using National Medical Services, Inc., including nicotine/cotinine testing, cannabis testing, and the expanded forensic panel in postmortem blood in order to cover any substances not tested.

### Statistical Analyses

The proportions of men with a manner of death of suicide were computed and compared using χ^2^ tests. To characterize the magnitude of group differences, an odds ratio (*OR*) was calculated for each analysis as an effect size (36) and interpreted according to widely used criteria (37), i.e., ORs between 1.20 and 1.71 = small, ORs between 1.72 and 2.40 = medium, and ORs >2.40 = large. Independent *t*-tests were used to compare groups based on age. Exploratory binary logistic regressions were used to model independent contributions of more than one variable. All statistical analyses were conducted using IBM SPSS Statistics 26.

## Results

From an initial sample of 202 men, there were 198, age 50 or greater, for whom their next-of-kin answered the question about their earlier in life participation in sports. The median age of the men at the time of death was 65.0 years (SD = 11.31, interquartile range = 57–75; range = 50–96). There were 34.8% of the men who participated in contact sports during high school (including football), and 29.8% participated in high school football. A personal history of traumatic brain injury was reported for 26.8% of the sample. Approximately one-third of the sample had suicide as their manner of death (34.8%). There was no significant difference in age between those with a manner of death of suicide vs. other causes [mean difference = −0.37 years; *t*_(196)_ = −0.221, *p* = 0.826]. There was no significant difference in age between those who played football and those who did not [mean difference = 0.15 years; *t*_(196)_ = 0.084, *p* = 0.933].

### Suicide

**Figure 1:**
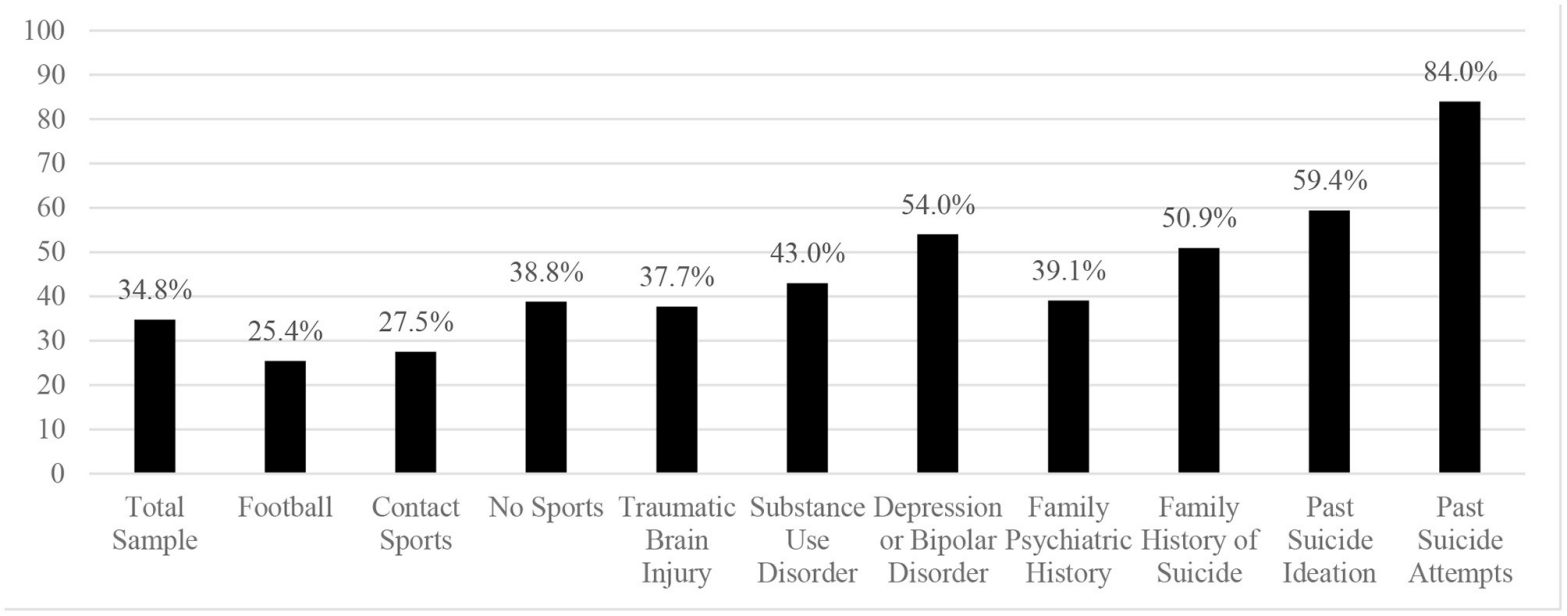
Suicide as a manner of death stratified by subgroups.

The proportions with suicide as a manner of death, by subgroup, are presented in Figure 1. There was no significant difference in the proportions of suicide between those who played football (25.4%) vs. those who did not (38.8%), or between those who played football and those who did not play any sports (38.8%) (see Table 2). There was no significant difference in the proportions of suicide between those who played contact sports (27.5%) vs. those who did not play contact sports (38.8%). There was also no significant difference in the proportions of suicide between those who had a history of TBI (37.7%) vs. those who did not (36.3%).

**Table 2:** Comparing binary subgroups on proportions with suicide as a manner of death and with a lifetime history of suicide attempts.

**Group comparison**	**Results**	**Effect size**
**Suicide**		
Football vs. no football	$\chi_{\left(1,\;198\right)}^{2}$ = 3.288, *p =* 0.070, OR = 0.537, 95% CI = 0.272–1.057	NS
Football vs. no sports	$\chi_{\left(1,\;188\right)}^{2}$ = 3.183, *p =* 0.074, OR = 0.539, 95% CI = 0.272–1.068	NS
Contact sports vs. no contact sports	$\chi_{\left(1,\;198\right)}^{2}$ = 2.494, *p =* 0.114, OR = 0.600, 95% CI = 0.318–1.134	NS
History of TBI vs. no history of TBI	$\chi_{\left(1,\;188\right)}^{2}$ = 0.034, *p =* 0.854, OR = 1.064, 95% CI = 0.551–2.052	NS
Depression/bipolar disorder vs. not	$\chi_{\left(1,\;181\right)}^{2}$ = 38.687, *p* < 0.001, OR = 10.712, 95% CI = 4.675–24.545	Very large
Substance use disorder vs. not	$\chi_{\left(1,\;183\right)}^{2}$ = 5.372, *p* < 0.020, OR = 2.075, 95% CI = 1.114–3.866	Medium
Prior history of suicide ideation vs. not	$\chi_{\left(1,\;168\right)}^{2}$ = 35.151, *p* < 0.001, OR = 8.038, 95% CI = 3.875–16.677	Very large
Prior history of suicide attempts vs. not	$\chi_{\left(1,\;177\right)}^{2}$ = 83.021, *p* < 0.001, OR = 36.422, 95% CI = 14.515–91.390	Very large
Family history of suicide vs. not	$\chi_{\left(1,\;179\right)}^{2}$ = 10.258, *p =* 0.001, OR = 2.927, 95% CI = 1.499–5.714	Large
Family psychiatric history vs. not	$\chi_{\left(1,\;175\right)}^{2}$ = 3.712, *p =* 0.054, OR = 2.098, 95% CI = 0.978–4.499	NS
**Suicide attempts**		
Football vs. no football	$\chi_{\left(1,\;177\right)}^{2}$ = 6.919, *p =* 0.009, OR = 0.335, 95% CI = 0.145-−0.776	Large
Football vs. no sports	$\chi_{\left(1,\;168\right)}^{2}$ = 6.320, *p =* 0.012, OR = 0.348, 95% CI = 0.149–0.810	Large
Contact sports vs. no contact sports	$\chi_{\left(1,\;177\right)}^{2}$ = 4.086, *p =* 0.043, OR = 0.471, 95% CI = 0.225–0.986	Medium
History of TBI vs. no history of TBI	$\chi_{\left(1,\;168\right)}^{2}$ = 6.903, *p =* 0.009, OR = 2.526, 95% CI = 1.253–5.092	Large
Depression/bipolar disorder vs. not	$\chi_{\left(1,\;166\right)}^{2}$ = 34.347, *p* < 0.001, OR = 16.309, 95% CI = 5.488–48.463	Very large
Substance use disorder vs. not	$\chi_{\left(1,\;168\right)}^{2}$ = 4.408, *p =* 0.036, OR = 2.084, 95% CI = 1.043–4.162	Medium
Prior history of suicide ideation vs. not	$\chi_{\left(1,\;164\right)}^{2}$ = 39.848, *p* < 0.001, OR = 10.724, 95% CI = 4.808–23.918	Very large
Family history of suicide vs. not	$\chi_{\left(1,\;174\right)}^{2}$ = 4.949, *p =* 0.026, OR = 2.180, 95% CI = 1.089–4.363	Medium
Family psychiatric history vs. not	$\chi_{\left(1,\;169\right)}^{2}$ = 0.617, *p =* 0.519, OR = 1.366, 95% CI = 0.626–2.979	NS

*NS, not significant; OR, odds ratio; CI, confidence interval*.

The proportions of men with suicide as their manner of death were compared based on the presence or absence of specific clinical characteristics. Those results are provided in Table 2. Those with the following clinical characteristics were significantly more likely to have suicide as their manner of death: (i) history of depression or bipolar disorder, (ii) history of substance use disorder, (iii) history of suicide ideation, (iv) history of suicide attempts, (v) family history of suicide.

### Suicide Attempts

**Figure 2:**
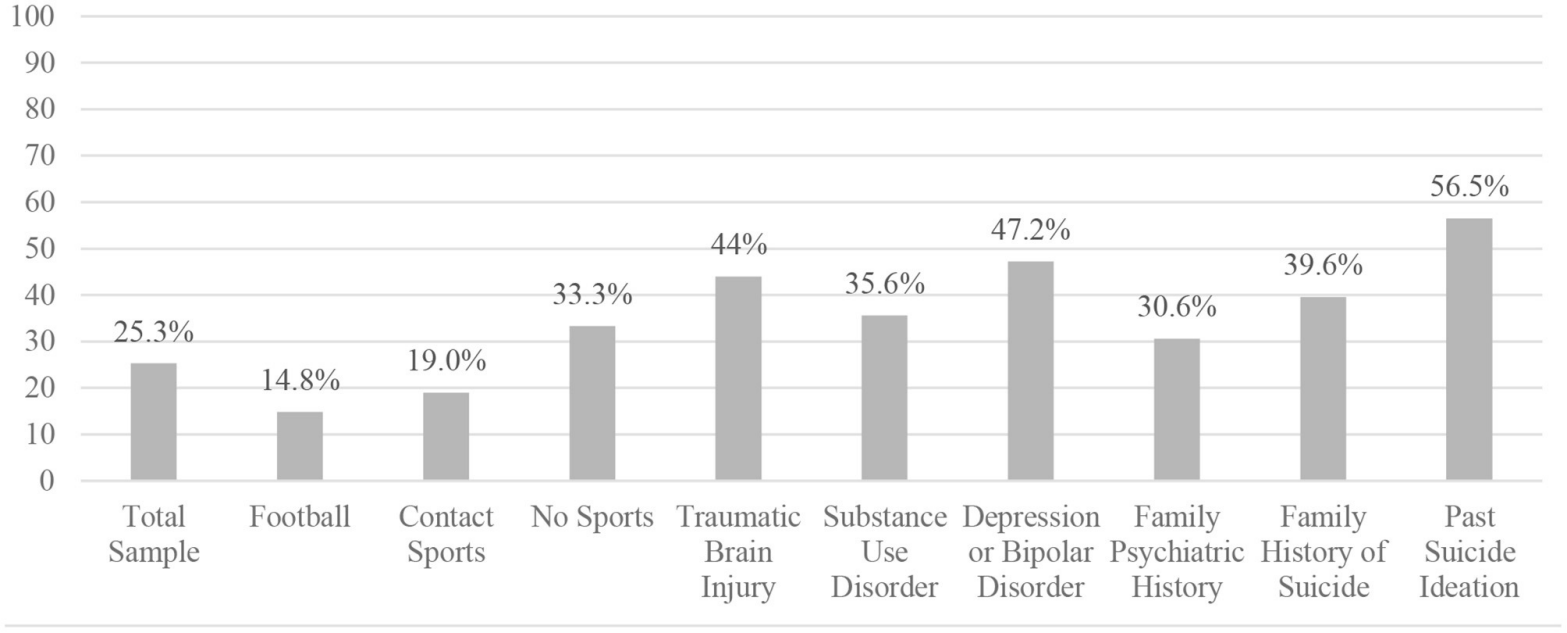
Proportions with a history of suicide attempts stratified by subgroups.

The proportions with suicide attempts, by subgroup, are presented in Figure 2. There was a statistically significant difference in the proportions with suicide attempts between those who played football (14.8%) vs. those who did not (34.1%), and between those who played football and those who did not play any sports (33.3%). In both cases, those who played football were *less* likely to have a lifetime history of a suicide attempt. There was also a significant difference in the proportions with suicide attempts between those who played contact sports (24.0%) vs. those who did not play contact sports (33.3%); those who played contact sports were *less* likely to have a lifetime history of a suicide attempt. Those with a history of TBI were significantly more likely to have a suicide attempt (44.9%) than those with no reported history of TBI (23.7%).

The proportions of men with a history of suicide attempts were compared based on the presence or absence of specific clinical characteristics. Those results are provided in Table 2. Those with the following clinical characteristics were significantly more likely to have a history of suicide attempts: (i) history of depression or bipolar disorder, (ii) history of suicide ideation, and (iii) family history of suicide.

Exploratory analyses were undertaken to examine further the association between a personal history of TBI and suicidality. As noted above, those with a history of TBI also had a greater history of suicide attempts. There was also a significant difference in the proportions with suicide ideation between those who had a history of TBI vs. those who did not [$\chi_{\left(1,\;159\right)}^{2}$ = 5.135, *p* = 0.023, OR = 2.213, 95% CI = 1.106–4.428, medium effect size]. There was not a significant difference in the proportions with documented depression or bipolar disorder between those who had a history of TBI vs. those who did not [$\chi_{\left(1,\;171\right)}^{2}$ = 1.186, *p* = 0.276, OR = 1.462, 95% CI = 0.737–2.903, large effect size]. There was a significant difference in the proportions with documented substance abuse disorders between those who had a history of TBI vs. those who did not [$\chi_{\left(1,\;173\right)}^{2}$ = 4.591, *p* = 0.032, OR = 2.106, 95% CI = 1.059–4.192, medium effect size].

Binary logistic regression was used to examine simultaneously TBI history and substance abuse history as predictors of suicidality. For suicide attempts, neither variable significantly contributed to the model when both were considered simultaneously [TBI B = 0.710, SE = 0.373, Wald = 3.621, *p* = 0.057, Exp (B) = 2.034, 95% CI = 0.979–4.226; substance use disorder B = 0.640, SE = 0.363, Wald = 3.106, *p* = 0.078, Exp (B) = 1.896, 95% CI = 0.031–3.862]. For suicide ideation, substance use disorder, but not TBI, contributed to the model when both were considered simultaneously [TBI B = 0.522, SE = 0.396, Wald = 1.742, *p* = 0.187, Exp (B) = 1.686, 95% CI = 0.776–3.663; substance use disorder B = 1.743, SE = 0.391, Wald = 19.883, *p* < 0.001, Exp (B) = 5.715, 95% CI = 2.656–12.297].

## Discussion

To our knowledge, this is the first study designed to examine whether participation in amateur football is associated with suicide in older adult men. There were no statistically significant differences in the proportions of suicide as a manner of death among those men with a personal history of playing football, or contact sports more broadly, compared to men who did not play football or who did not play sports. Men who played football, and men who played contact sports, were significantly *less* likely to have a lifetime history of a suicide attempt.

A personal history of traumatic brain injury was not associated with suicide, but those with a history of TBI were more likely to have suicide ideation and a suicide attempt. Those with a history of TBI were not more likely to have a diagnosis of a mood disorder, but they were more likely to have a documented substance abuse disorder. Controlling for substance use disorders, there was no significant independent association between TBI history, suicide ideation, or suicide attempts. Men with mood disorders, substance use disorders, and those with a history of suicide ideation or attempts were more likely to have suicide as a manner of death. Moreover, those men with a family history of suicide were more likely to have prior suicide attempts and to have completed suicide. All of those factors are well-established risk factors in the literature (27, 31–33, 38).

### Depression and Suicidality in Former Amateur Football Players

The results of this study are consistent with six prior studies relating to participation in high school football, depression, suicidality, and suicide. Three large-scale studies, using the *same* longitudinal database, have found that men who played high school football, compared to those who did not play sports, did not have greater rates of depression or suicidality in their early or late 20s (20–22). A survey study of middle-aged men who played high school football reported that they did not have higher lifetime rates of being prescribed medication for anxiety or depression or receiving treatment from a mental health professional, and they did not endorse greater depression, anxiety, or anger over the past year (39). A large-scale longitudinal study of older adult men, with an average age of 65, revealed no association between playing football in high school and current problems with depression (23).

Very little is known about suicide in middle-aged or older adult men who played high school football. A recently published study (40) provides information relating to the long-term mental health of former amateur football players in the online Supplementary Material. The study was focused on CTE neuropathologic change, but the unpublished online Supplementary Material provided extensive information about the mental health of these men. These researchers used autopsy cases from the Mayo Clinic Tissue Registry. They relied on primary historical obituary and yearbook records to identify and compare former athletes to people who did not participate in sports, and a medical records-linkage system to examine billing codes relating to various psychiatric and neurological diagnoses. Using the frequency data presented in their online Supplementary Table 3, we statistically compared the 140 men who played football to the 245 men who did not play sports. The former football players had significantly *lower* rates of depression [25.0% vs. 37.1%, $\chi_{\left(1\right)}^{2}$ = 5.97, *p* = 0.015, odds ratio (OR) = 1.77, 95% confidence interval (CI) = 1.12–2.81], and there was no statistically significant difference in their rate of suicide [3.6% in those who played football vs. 8.2% in those who did not play sports; $\chi_{\left(1\right)}^{2}$ = 3.094, *p* = 0.079].

### Suicide in Former Professional Football and Soccer Players

It is important to note that there are large scale studies illustrating that former NFL players are at *lower* risk for suicide than men from the general population (12, 13). Lehman and colleagues (13) examined suicide mortality in a cohort of 3,439 NFL players who played between 1960 and 2013 and identified 12 who had died by suicide (0.3% of the cohort). A total of 537 had died during the observation period, so 2.2% had suicide as their manner of death. The expected number of deaths by suicide from men in the general population was more than double the number in former players (i.e., 25.6), resulting in a standardized mortality ratio of 0.47 (95% CI = 0.24–0.82) (13). Relatedly, there was no significant difference in the rate of suicide as a manner of death or the age at which a person completed suicide between former professional soccer players and their matched controls (41). In that study, suicide was recorded as the manner of death in 19 (0.25%) of 7,676 former professional soccer players and 93 (0.40%) of 23,028 population control subjects. Of those who had died during the 18-year observation period, the rate of death by suicide was 1.6% (19/1,180 deaths) for the former soccer players and 2.4% (93/3,807 deaths) for the matched control subjects.

### Suicide in Men From the General Population

Suicide is a leading cause of death in adolescents and young adults, and men are three times more likely to complete suicide than women (27). In 2016, the incidence of suicide in men between the ages of 15 and 19 was estimated to be 14.8/100,000, and for those between 20 and 24 it was 26.8/100,000. For men between the ages of 55 and 64, the incidence was 28.5/100,000, and for those between the ages of 75 and 84 it was 33.6/100,000 (27). The causes of suicide are usually complex, multifactorial, and difficult to predict or understand in individual cases.

Suicidality is a clinical feature of depression. Rates of suicidality and suicide also are higher in people with anxiety disorders (42). People who experience major depressive disorders are at considerable risk for attempting suicide (24), and people with psychotic or delusional depression are at even greater risk for attempting suicide (43). For people who are experiencing depression, some risk factors for completing suicide include male gender, family history of a psychiatric disorder, previous attempted suicide, more severe depression, hopelessness, comorbid anxiety, and misuse of alcohol or drugs (44). People who misuse alcohol (28), drugs (29), benzodiazepines (45), and opioids (46, 47) are at increased risk for suicidal ideation. Psychological autopsy studies indicate that drug use disorders are strong *proximate* risk factors for completed suicide (30).

In adults, all different types of childhood maltreatment including sexual abuse, physical abuse, and emotional abuse are associated with increased risk for depression and suicidality (48, 49), and there is a statistically significant association between lifetime history of sexual abuse and a lifetime diagnosis of anxiety disorder, depression, post-traumatic stress disorder, sleep disorders, and suicide attempts (50). In older adults, suicidal behavior is associated with functional disability and specific medical problems such as malignant diseases, neurological disorders, pain, arthritis, chronic obstructive pulmonary disease, and liver disease (51). In older adults, moderate to severe pain is associated with increased risk for suicidal ideation and attempts; arthritis, back/neck problems, and headaches are associated with higher risks of suicidal behavior; and pain is a much stronger predictor for suicide in men than in women (52). Alzheimer's disease also is associated with a risk of suicide (53).

A CDC report examining suicides identified common precipitating events as depression, substance abuse, a life crisis, intimate partner problems, physical health problems, occupational or financial problems, and criminal or legal proceedings (27). These circumstances, in a sample of more than 18,000 men who completed suicide, are summarized in Table 3. High risk patterns of thinking, or cognitive states, associated with suicidality include hopelessness (54, 55), problem-solving deficits, such as difficulty conceptualizing adaptive solutions to life problems (56), and perceived burdensomeness and thwarted belongingness (a belief that one does not have meaningful interpersonal relationships) (57). Mental pain, considered distinct from depressogenic negativism, is characterized by a person's perception of strong negative feelings and changes in one's sense of self and functioning. It is conceptually related to grief. Mental pain is considered a strong vulnerability factor for suicidal ideation (58).

**Table 3:** Circumstances preceding suicide in men from the US general population.

**Circumstance**	**Percentage**
Current depressed mood	37.2%
Alcohol problem	18.5%
Substance abuse problem (excludes alcohol)	16.2%
Physical health problem	22.2%
Intimate partner problem	29.0%
Crisis during previous or upcoming 2 weeks	32.3%
Argument or conflict	16.1%
Job problem	11.2%
Financial problem	9.2%
Recent criminal legal problem	9.8%
History of suicidal thoughts or plans	31.6%
History of suicide attempt	16.5%

*N = 18,138. The circumstances preceding death are defined as the precipitating events that contributed to the suicide. The actual rates are likely higher because the source of this information is derived entirely from coroner/medical examiner and law enforcement investigative reports (vs. other sources, such as family interviews or reviews of medical records) (27)*.

### Limitations

There are four important methodological issues to consider when interpreting the primary results of this study, the null findings relating to suicidality and participation in football or contact sports. First, information about participation in contact sports was provided during an interview of the next-of-kin. It was not verified using other sources, such as school yearbooks. Second, we had no information relating to how much exposure to football the decedents had, or how many concussions they might have sustained while playing football or over the course of their lives. Third, by design, we studied men aged 50 and over—so the results do not generalize to men under the age of 50. Finally, the sample we used, without question, is not representative of men from the general population who played football, including the fact that there were virtually no men who were Black or African American in the sample. This sample has major ascertainment bias inherent in the nature of the brain donation program—individuals being referred for autopsy to investigate cause of death, many of whom have a known history of psychiatric illness and/or substance abuse. However, it is the very nature of this program that allowed us to have a sufficient sample size of men with suicide as their manner of death to complete this study.

Personal history of TBI was examined in this study as an exploratory aim. There are major limitations associated with this variable. The variable is based on information from a family member, and the accuracy of this was not independently verified through medical records. The severity of injury could sometimes be inferred, such as descriptions involving several concussions in sports, multiple skull fractures and intracranial bleeding after a fall from a height, a period of coma following a motorcycle accident, or need for neurosurgery to evacuate a subdural hematoma after a fall—but for most cases we could not determine severity of injury. These results, therefore, should be interpreted with caution and replication with a larger sample would increase confidence in these findings.

### Conclusions

In the present study, suicide attempts and completed suicide were not related to participation in amateur football or contact sports. Suicide was, however, related to well-established risk factors such as a personal history of a mood disorder, substance abuse disorder, prior suicide ideation, suicide attempts, and a family history of suicide attempts. In conclusion, this study adds to a steadily growing body of evidence suggesting that playing high school football is not associated with increased risk for suicidality or suicide during adulthood.

## Data Availability Statement

The original contributions presented in the study are included in the article, further inquiries can be directed to the corresponding author. The statistical code, syntax, output, and analyses are available to qualified researchers upon request. Requests to access the information should be directed to Grant L. Iverson, giverson@mgh.harvard.edu.

## Ethics Statement

The use of data from the decedents was approved by the Western Michigan University Homer Stryker M.D. School of Medicine. The subjects were obtained from the Lieber Institute for Brain Development (LIBD) brain donation program. Postmortem brain samples were donated to the LIBD from the Office of the Medical Examiner of the Western Michigan University Homer Stryker M.D. School of Medicine, Department of Pathology (WIRB protocol #1126332), with the informed consent of legal next-of-kin at the time of autopsy.

## Author Contributions

GI conceptualized and designed the study, conducted the literature review, conceptualized the statistical analyses, wrote the first draft of the manuscript, and agrees to be accountable for the content of the work. AD-S completed team-based clinical diagnostic reviews for each case, managed all the data, participated in conceptualizing the study, reviewed drafts of the manuscript, and agrees to be accountable for the content of the work. TH and JK administratively manage the brain bank, conducted interviews with next-of-kin, completed team-based clinical diagnostic reviews for each case, participated in conceptualizing the study, reviewed drafts of the manuscript, and agree to be accountable for the content of the work. BE reviewed and selected appropriate autopsy cases for referral to for the brain donation program, helped design the database, edited drafts, and agrees to be accountable for the content of the work. AF-H edited drafts and agrees to be accountable for the content of the work. JdJ serves as the site principal investigator for the brain donation program, wrote portions of the manuscript, edited drafts, and agrees to be accountable for the content of the work. RC assisted with conceptualizing the study, wrote portions of the manuscript, edited drafts, and agrees to be accountable for the content of the work. All authors contributed to the article and approved the submitted version.

## Funding

GI acknowledges unrestricted philanthropic support from ImPACT Applications, Inc., the Mooney-Reed Charitable Foundation, the National Rugby League, and the Spaulding Research Institute. These entities were not involved in the study design, collection, analysis, interpretation of data, the writing of this article or the decision to submit it for publication.

## Conflict of Interest

GI serves as a scientific advisor for NanoDX^®^, Sway Operations, LLC, and Highmark, Inc. He has a clinical and consulting practice in forensic neuropsychology, including expert testimony, involving individuals who have sustained mild TBIs (including former athletes), and on the topic of suicide. He has received research funding from several test publishing companies, including ImPACT Applications, Inc., CNS Vital Signs, and Psychological Assessment Resources (PAR, Inc.). He has received research funding as a principal investigator from the National Football League, and subcontract grant funding as a collaborator from the Harvard Integrated Program to Protect and Improve the Health of National Football League Players Association Members. RC is subcontracted to the Lieber Institute for Brain Development to assist with brain examinations. He has received research funding from the Chuck Knoll Foundation for Brain Injury Research for brain studies that were not included in this manuscript. A small percentage of his practice involves forensic neuropathology, including expert testimony, some of which involves former contact sport athletes. The remaining authors declare that the research was conducted in the absence of any commercial or financial relationships that could be construed as a potential conflict of interest.

## Publisher's Note

All claims expressed in this article are solely those of the authors and do not necessarily represent those of their affiliated organizations, or those of the publisher, the editors and the reviewers. Any product that may be evaluated in this article, or claim that may be made by its manufacturer, is not guaranteed or endorsed by the publisher.
